# Conventional and Novel Diagnostic Tools for the Diagnosis of Emerging SARS-CoV-2 Variants

**DOI:** 10.3390/vaccines11020374

**Published:** 2023-02-06

**Authors:** Vivek P. Chavda, Disha D. Valu, Palak K. Parikh, Nikita Tiwari, Abu Sufiyan Chhipa, Somanshi Shukla, Snehal S. Patel, Pankti C. Balar, Ana Cláudia Paiva-Santos, Vandana Patravale

**Affiliations:** 1 Department of Pharmaceutics and Pharmaceutical Technology, L. M. College of Pharmacy, Ahmedabad 380009, Gujarat, India; 2 Formulation and Drug Product Development, Biopharma Division, Intas Pharmaceutical Ltd., 3000-548 Moraiya, Ahmedabad 380054, Gujarat, India; 3 Department of Pharmaceutical Chemistry and Quality Assurance, L. M. College of Pharmacy, Ahmedabad 380009, Gujarat, India; 4 Department of Pharmaceutical Sciences and Technology, Institute of Chemical Technology, Mumbai 400019, Maharashtra, India; 5 Department of Pharmacology, Institute of Pharmacy, Nirma University, Ahmedabad 382481, Gujarat, India; 6 Pharmacy Section, L. M. College of Pharmacy, Ahmedabad 380009, Gujarat, India; 7 Department of Pharmaceutical Technology, Faculty of Pharmacy of the University of Coimbra, University of Coimbra, 3000-548 Coimbra, Portugal; 8 REQUIMTE/LAQV, Group of Pharmaceutical Technology, Faculty of Pharmacy of the University of Coimbra, University of Coimbra, 3000-548 Coimbra, Portugal

**Keywords:** COVID-19, variant of concern, variant, diagnosis, detection, nano-diagnosis, SARS-CoV-2, nanotechnology, exosome

## Abstract

Accurate identification at an early stage of infection is critical for effective care of any infectious disease. The “coronavirus disease 2019 (COVID-19)” outbreak, caused by the virus “Severe Acute Respiratory Syndrome Coronavirus 2 (SARS-CoV-2)”, corresponds to the current and global pandemic, characterized by several developing variants, many of which are classified as variants of concern (VOCs) by the “World Health Organization (WHO, Geneva, Switzerland)”. The primary diagnosis of infection is made using either the molecular technique of RT-PCR, which detects parts of the viral genome’s RNA, or immunodiagnostic procedures, which identify viral proteins or antibodies generated by the host. As the demand for the RT-PCR test grew fast, several inexperienced producers joined the market with innovative kits, and an increasing number of laboratories joined the diagnostic field, rendering the test results increasingly prone to mistakes. It is difficult to determine how the outcomes of one unnoticed result could influence decisions about patient quarantine and social isolation, particularly when the patients themselves are health care providers. The development of point-of-care testing helps in the rapid in-field diagnosis of the disease, and such testing can also be used as a bedside monitor for mapping the progression of the disease in critical patients. In this review, we have provided the readers with available molecular diagnostic techniques and their pitfalls in detecting emerging VOCs of SARS-CoV-2, and lastly, we have discussed AI-ML- and nanotechnology-based smart diagnostic techniques for SARS-CoV-2 detection.

## 1. Background

SARS-CoV-2 is the seventh member of the coronavirus family to spread in humans, and it is blamed for the current COVID-19 pandemic. The level of dissemination of these previous variants was relatively limited, in contrast to SARS-CoV-2 [1,2]. SARS-CoV was first detected in 2002 and had a mortality of 10%, infecting 8000 people, but was suppressed by 2004. In 2012, another coronavirus family member emerges, namely, Middle East respiratory syndrome coronavirus, which had a mortality of roughly around 30% [3]. Multiple pneumonia cases with ambiguous etiologies were discovered in China in the middle of December 2019; these cases were subsequently identified as being caused by a novel coronavirus in early January 2020. The virus was finally confirmed to be the cause of COVID-19 and given the name SARS-CoV-2 [4,5,6]. The virus spread quickly around the world despite considerable, extensive attempts to confine the illness in China, and the WHO declared COVID-19 a pandemic in March 2020 [7,8]. The virus has further evolved itself into various genera such as alpha, beta, gamma, delta, omicron, deltacron, and many more bases on the mutation, mainly influenced by the spike protein.

## 2. The Genetic Makeup of SARS-CoV-2

SARS-CoV-2 genome comprises 14 ORFs (open reading frames) that encode 27 viral proteins [9]. The ORF region (ORF1ab) of the genome also encodes for the enzyme RNA-dependent RNA polymerase (RdRp), which is crucial for replication of virus [10]. The major structural proteins encoded by the viral genome are the spike (S) glycoprotein, small envelope (E) protein, matrix (M) protein, and nucleocapsid (N) protein [11]. The S1 domain of the spike glycoprotein aids in ACE2 (angiotensin-converting enzyme 2) receptor binding, while the S2 protein facilitates the cell membrane fusion [12]. The new SARS-two CoV-2’s key traits are its enhanced capacity to attach to human ACE2 receptors specifically and the existence of a functional polybasic (furin) cleavage site at the interface between the S1 and S2 subunits of its spike glycoprotein, which allows the virus to bind and enter host cells [13]. Among coronaviruses, the receptor-binding domain (RBD) located in the spike protein is the most variable component and determines the affinity by which the virus binds to the ACE2 receptors on the host cell surface [14]. Unfortunately, SARS-CoV-2 appears to have a high binding affinity with the ACE2 receptors expressed on human cells [15]. The presence of a polybasic cleavage site at the S1–S2 junction of spike protein paves the way for proteases, including furin, to ultimately define the extent of viral introduction into the host cells [14].

## 3. Sampling of SARS-CoV-2

In the general consideration, diagnosis relies on the sample collection and the respective organ. It is crucial to select specific tissue or organ in order to attain precise detection, which helps in the early and constructive treatment approaches. On the normal bases, nasopharyngeal swabs (NPS) are incorporated as a sample collection method for COVID-19, whereas other methods such as oropharyngeal swab, saline, and NP wash function as a target of upper respiratory tract, wherein lower respiratory tract sampling system involves the sampling from sputum, bronchoalveolar lavage fluid (BLF), tracheal spirates, and many more [16]. Tapia et al. conducted a comparative study between NPS, Nasal mid-turbinate swab (NMTS), and saliva samples using quantitative RT-PCR as diagnostic kit [17]. It was observed that the number of positive samples for NPS, saliva, and NMTS were 96.4%, 85.7%, and 78.6%, respectively. It provides clinical evidence for the preferential use of NPS [17]. For the serological detection, many antigens detection diagnostic tests are employed. Antigen detection tests are also performed in an infected person’s urine sample [18]. However, the hurdle in employing this method as diagnostic standard is its low sensitivity [19]. Both oropharyngeal and nasopharyngeal specimen are collected in an single tube in order to maximize the sensitivity while anterior nasal specimen incorporates the use of the same swab in both the nostrils [20]. A comparative study on the sampling efficacy is described in Table 1.

## 4. SARS-CoV-2 Diagnostic Techniques and Their Clinical Relevance

Timely diagnosis is the most important strategy employed to prevent the spread of COVID-19. Nucleic acid-based testing remains the gold standard for SARS-CoV-2 testing [22]; however, owing to the issues related to capacity, sensitivity, time efficiency, and affordability, the demand for the development of effective alternative methods of diagnosis to overcome these concerns has risen [23]. In the coming few sections of this review, we will discuss different available techniques used for the diagnosis of SARS-CoV-2.

### 4.1. Nucleic Acid-Based Tests

**Figure 1:**
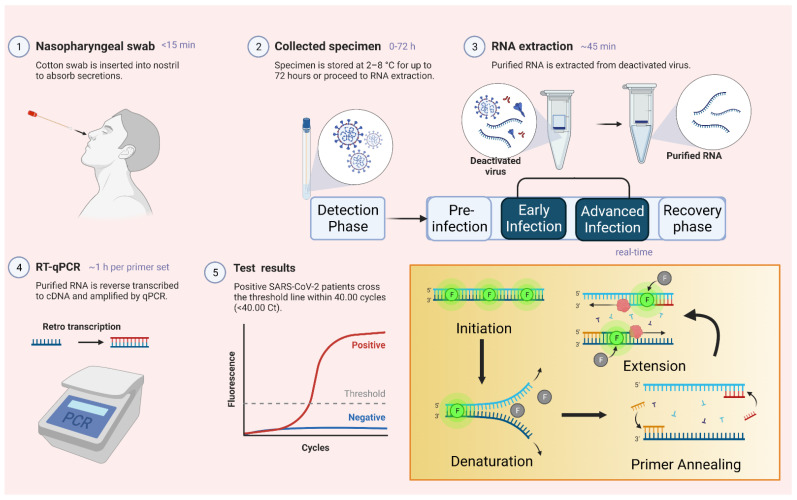
Methodology for RTPCR testing of COVID-19.

“Reverse transcription-polymerase chain reaction (RT-PCR)” is presently considered the “gold standard” for the detection of SARS-CoV-2 infection. The test is based on the identification and amplification of the viral genetic material [24]. This method involves the formation of complementary DNA (cDNA) from SARS-CoV-2 RNA through reverse transcription and subsequent amplification of specific cDNA regions [22]. The procedure broadly comprises four steps: (a) collection and processing of samples taken from suspected individuals usually through nasopharyngeal swabs; (b) extraction of RNA from the isolated sample; (c) reverse transcription from cDNA; (d) amplification and detection [10]. As per the protocol shared by the WHO, in early 2020, primers targeting the E gene and RNA dependent RNA polymerase gene (for specificity) are used to distinguish between SARS-CoV-2 and the previously known SARS-CoV [25]. A study conducted by Anantharajah et al. stated that while using the primer and probes as per the guidance of WHO influence the diagnosis property of RT-PCR. A significant difference in sensitivity was observed on the pioneer of different primers and probes [26]. Several other primers have also been developed that target the ORF1 and N genes by researchers in China, Hong Kong, and Japan [10,27,28], while in the USA, the “Food and Drug Administration (FDA)” published its protocol for RT-PCR based on primers targeting the N gene (Figure 1) [10,29].

Despite being regarded as the most reliable technique for virus detection, instances of poor performance are reported regarding the sensitivity in the case of RT-PCR [30]. Incidences of false positivity are also reported. In one case report, which was published in January 2021, a patient confirmed COVID-19 positive based on his RT-PCR test had normal computed tomography (CT) findings and repeatedly tested negative for antibody (IgG) [31]. On the contrary, one systematic review suggests the possibility of initial false-negative results based on their findings, concluding that up to 54% of initial COVID-19 testing may end up showing false-negative results. Accordingly, repeated confirmatory tests are recommended [32,33]. Sample collection with detectable levels of viral RNA is one of the most critical steps when RT-PCR testing is performed, the failure of which leads to false results [22]. Keeping in mind the compromised sensitivity of the test, it is apparent that RT-PCR alone is not completely reliable. As a result, complementary tests (as CT scan findings, serum-based testing) in combination with RT-PCR results can be beneficial in confirming the COVID-19 infection [34]. Along with a high degree of effectiveness, it ensures a pocket-friendly diagnosis. With it being effective, it is cost effective as well, which adds to its benefits. In the time of the pandemic, a shortage of reagents for extraction was aroused, which was overcome by alternative options [35].

A study on 225 adults was carried out in order to compare RT-PCR with the viral culture. The basis of criteria was antigen sensitivity test. The sensitivity for RT-PCR and viral culture was 64% and 84%, respectively [36]. These stated the superior sensitivity of viral culture as compare to RT-PCR. Another study was conducted of RT-PCR in comparison with Cobas 6800. It was noted that Cobas 6800 even detected eight patients (total 188) who had been stated negative by RT-PCR. The false negative result of RT-PCR occurred because the RdRp and N genes of SARS-CoV-2 remained undetected [37]. A study on diagnostic effectiveness stated that RT-PCR had an 84% positive predictive value (PPV) for positive tests and 32.4% false-positive reports [38]. This data suggested how reliable diagnostic using RT-PCR can be.

### 4.2. Computed Tomography (CT) Scanning

The non-invasive chest CT scans provide the X-ray measurements of infected individuals taken from different angles to generate cross-sectional images. These images are then evaluated by qualified radiologists to find the abnormalities or severity in the lungs of the infected patients [39]. The images of CT scans differ as the infection progresses in the lungs. The most commonly witnessed lung changes during the COVID-19 infection is the appearance of bilateral and peripheral ground-glass opacities that generally appear during 0–4 days of infection. With disease progression, the irregularly shaped stone-like pattern appears in the chest scan accompanied by lung consolidation [40,41]. CT scanning tends to have high sensitivity (86–98%) and reduced the chances of false-negative results [42]; however, the specificity of the test is still an issue, as the results may coincide with other viral pneumonia [42]. Further clinical correlations and confirmatory testing are warranted when CT scans are used as the diagnostic tools for SARS-CoV-2 detection. When comparing with RT-PCR, the cost per utility was higher for CT scanning which conclude the limitation of it. A comparative study was conducted corresponding to diagnostic property of CT scan and RT-PCR. It was concluded that the former is less prone to giving a false-negative result (9%) as compared to latter [43]. Another clinical study suggested that the proportion of false-positive CT scans was 7.2% [44]. Generally, RT-PCR tests are preferred over CT scan for the early detection of COVID-19 [45].

### 4.3. Protein-Based Tests

Protein-based tests rely on their ability to detect the proteins, glycans, or antibodies that are peculiar to a particular virus [46]. These tests are based on the principles of antigen–antibody interactions, where either the antigen or the antibody from the patient’s sample reacts with the respective antibody or antigen present on the test kit [47]. The major challenges faced when the diagnosis of coronavirus is carried out by RT-PCR are the limited capacity, irregular supply, delay in results, and insufficient expertise to handle the technique [48]. On the contrary, protein-based tests including antigen-based tests are easily available, rapid, and do not require specific expertise to perform the diagnosis.

For antigen-based tests, different detection kits are designed by the researchers that detect different antigen proteins in the patient’s sample. The majority of these test kits are based on the detection of spike proteins, glycan proteins, and nucleocapsid proteins that are peculiar to SARS-CoV-2 [46,49]. More importantly, the antigen-based tests appear to have high sensitivity that gradually decreases as the days pass. Accordingly, the antigen-based test showed high sensitivity (80%) during the first 7 days of infection, followed by 76% in the second week, and a mere 19% in the third week, when data was compared with PCR. On the contrary, antibody-based tests showed the opposite trend in terms of sensitivity such that testing during the initial infection phase had only 26.8% sensitivity, which increased to 76% by the 14th day of infection [50]. Antigen test detects viruses when they are in their most virulent form wherein antibody test detects them as soon as the defense mechanism is provoked [51]. In the normal healthcare practice, antibody test is performed only after the recovery of the patients [52]. In one similar study, the highest sensitivity (95–100%) of antibody-based test kits developed by different manufacturers was observed during the fourth week of infection [53]. Again, the sensitivity of the antigen-based test was found to be maximum during the first week of infection, owing to high viral loads during the initial days [54]. Table 2 summarizes the prominent diagnostic techniques for the detection of coronavirus.

Antibodies are incorporated in order to attain rapid, simple results with the greatest sensitivity. IgM provides with a first line defense against many viral diseases [62]. Even with the wide acceptability of IgG and IgM based tests, it has its pitfalls. They are effective after a time lapse of 5–10 days after the appearance of symptoms [63]. Numerous other diagnostic methods are used such as RT-PCR, CRISPR/Cas, antigen test and many more. Among them, lateral flow immunoassay (LFIA) method is preferred because of its selectivity, quick response, etc. It is capable of detecting IgG and IgM within 15 min [58,64]. Along with the quick response, it has acceptable sensitivity and specificity (88.66% and 90.63%, respectively) [64]. Being a great method for diagnosis, yet it owns its drawbacks as well. False negatives commonly occur due to various reasons such as multi-step procedure, irrational immobilization of proteins, unequal protein–probe conjugation and many more [65]. A study was conducted in order to check the false-positive result of IgG-mediated diagnosis kit using RIAT. It concluded that out of total patients under supervision, 57% gave a false-positive test. They actually suffered from human common cold coronavirus pneumonia [66]. This states the higher sort of variability of result.

In antibody detection tests, two immunoglobulins, namely IgG and IgM, are usually detected, and appear variably during infection, showing several diagnostic values in multiple studies [67,68]. The levels of IgM increase in the first week, following SARS-CoV-2 exposure; however, the IgG starts rising from the second week, and lasts for a longer duration, although IgM levels may subside in a long run. Another immunoglobulin, IgA, appears within 4–10 days of infection [47]. As a result, the serum levels of these immunoglobulins at different times can be a predictor of the disease stage. Novel diagnostic kits detecting the combination of these antibodies in blood samples are, therefore, developed to improve the sensitivity and specificity of the diagnosis. A rapid point of care immunoassay developed by Li et al. to simultaneously measure the levels of IgG and IgM in blood samples showed improved sensitivity and specificity when clinically evaluated [58]. Similarly, the sensitivity and specificity of the test were improved in another study that used kits with combined efficiency to detect IgA, IgG, and IgM [69].

### 4.4. CRISPR/Cas System

“Clustered regularly interspaced short palindromic repeats (CRISPR)/CRISPR-associated gene (Cas) (CRISPR/Cas)” is a powerful technique of gene editing that is avidly employed in different fields of science. Importantly, the DNA/RNA targeting capacity of CRISPR can be employed in conjunction with the amplification process for the simultaneous detection and quantification of the viral load in the isolated samples [59]. For the detection of SARS-CoV-2, the CRISPR technique offers a rapid, highly efficient, portable, and low cost per sample evaluation [70]. Mammoth Biosciences and Sherlock Biosciences are the pioneer companies that have independently developed CRISPR-based diagnostic kits for COVID-19 detection. The detection kit developed by Mammoth Biosciences, i.e., “DETECTR (DNA endonuclease-targeted CRISPR trans-reporter)” utilizes the CRISPR-Cas12-based lateral flow system to target N and E genes. On the other hand, Sherlock Biosciences developed “SHERLOCK (specific high sensitivity enzymatic reporter unlocking)”, which targets the S gene and Orf1ab of SARS-CoV-2 [60]. More recently, an upgraded version of SHERLOCK (SHERLOCKv2) was developed to make the simultaneous detection of more than one target gene sequence possible [71]. CRISPR-based SARS-CoV-2 diagnostic tools utilize the Cas endonucleases for their collateral cleavage activity. The CRISPR/Cas system-associated effector complexes work by locating and subsequent binding to the target gene (guided by the CRISPR RNA). The binding takes place at the location that resembles the spacer sequence of crRNA (CRISPR RNA) that usually lies near the short sequence called protospacer adjacent motif (PAM) or protospacer flanking site (PFS) [72]. The sensitivity of the CRISPR/Cas-based systems is comparable to the conventional RT-PCR. A CRISPR-based diagnostic kit (CRISPR-COVID-19) developed by Hou et al. [73] had single-copy sensitivity, high specificity, and was time-efficient with the ability to produce results in 40 min. Another CRISPR-based system developed and evaluated by Huang et al. [74] utilized the CRISPR Cas12a/gRNA complex accompanied by a fluorescent probe. The system showed highly specific detection of SARS-CoV-2 with sensitization efficiency of two copies per sample in a time duration of 50 min. Interestingly, the results were comparable with the standard quantitative RT-PCR [74]. CRISPR-based diagnosis has advantages over RT-PCR, such as thermocycling, single-nucleotide specificity, integration with accessibility, lateral flow strips and many more. With these advantages, CRIPSR is presumed to give rapid, visual detection and sensitivity towards SARS-CoV-2 [61]. With the benefits it holds, there are certain drawbacks as well. The off-target effect of CRISPR is one of the greatest hurdles. Nucleic acids interact with 3–5 mismatch targets, which raises concern as it is actively involved in diagnosis and treatment and can lead to false prediction. Initial screening of the off-target effect can be performed by tools such as DISCOVER, Digenome-Seq, SELEX and many more [75]. Research suggested that off-target delivery occurs because the force of DNA rehybridization was lower in comparison with the affinity of Cas9 towards nontarget DNA strands. Taking this hypothesis as a base, scientist found two mutants, S845K and L847R, which reduce the selectivity [76]. To overcome this pitfall, various methodologies are employed. Structural modification can be performed in order to introduce a novel mutation (Mut268) in *Staphylococcus aureus* (SaCas9), which helps to maintain the effectiveness of protein with a reduced off-target effect [77].

## 5. Limitations of the Current Testing Tools

The collection of respiratory tract samples during the preanalytical stage, in the right period and from the appropriate anatomic site, is critical for a rapid and precise molecular diagnosis of COVID-19. Regular testing can be of significance if a patient has clinical evidence of viral pneumonia, radiographic findings consistent with COVID-19 pneumonia, and/or potential contact history. Similarly, it is difficult to decide how the outcomes of one unnoticed result could influence decisions about patient quarantine and social isolation, particularly when the patients themselves are health care providers [78,79].

### 5.1. Detecting and Observing Patients with Severe COVID-19 Pneumonia Symptoms at a Late-Stage

Lower respiratory tract specimens should ideally be assembled using sputum or bronchoalveolar lavage, as they provide the maximum viral antigens for diagnostic purposes [80]. It was recently observed that bronchoalveolar lavage (BAL) fluid samples produced the highest SARS-CoV-2 RNA antigen, even though this observation did not have any comparative data to be evaluated against the nasopharyngeal swab (NP) swabs [81].

### 5.2. Assay Selection

The recently developed rapid point-of-care immunoassays for SARS-CoV-2 detection are typically lateral flow assays, but now high-throughput immune analyzer forms for population-level screening are also being developed [82]. Based on previous experience with influenza (flu) viruses, the lateral flow assays give the advantage of low cost and quick detection time but face a major drawback due to their poor performance in early-stage detection [83]. Monoclonal antibodies directed specifically against SARS-CoV-2 antigens are being developed. However, there is a concern that, due to the high rate of mutation in the antigens in COVID-19 patients, the detection system may miss cases, due to low antigen load or inconsistency in sample processing methods. IgM responses are undeniably generic, and it takes weeks for specific IgG responses to develop. In the continuation of the disease, it is not effectively utilized in the management of cases.

### 5.3. Random Amplification

Deep-sequencing technologies were used majorly for the documentation of SARS-CoV-2 earlier. Next-generation sequencing and metagenomic next-generation sequencing approaches of deep-sequencing will continue to play a role in identifying future SARS-CoV-2 mutations, besides being currently unsuitable for diagnosing COVID-19 [84].

Finally, the importance of quick advancement in the development of integrated, random-access, point-of-care molecular devices for the precise detection of SARS-CoV-2 infections cannot be overstated [85]. These rapid-turnaround diagnostics will be critical for the treatment of patients in real time, and for deciding on the choices one has to make for infection control, especially when there are other less infectious mutagenic forms of pneumonia present, and care supplies related to it are limited [86]. These tests are safe, straightforward, quick, and they can easily be performed in local hospitals and clinics that are already equipped with the necessary instruments and are in process of detecting and curing such patients [87,88].

## 6. Viral Variants and Diagnosis

Sequencing data can be used to determine if present diagnostic tests are still valid, as well as to keep track of the development of novel diagnostics. Virus mutations can impair the precision of diagnostic tests [89,90]. Researchers can identify any mutations of particular relevance by analyzing sequencing data regularly, which can then be investigated to see if they affect test function [91,92]. Alternatively, if a test does not appear to be used as expected, such as producing false-negative findings regularly, samples could be sequenced to check if they include a mutation that causes test failure [93,94]. Routine SARS-CoV-2 testing can be classified by the test target, with direct detection of viral RNA or antigens, or indirect detection of anti-SARS-CoV-2-specific antibodies indicating current or history of illness [95] (refer to Table 3). Primers (short DNA sequences) are used in nucleic acid amplification tests (NAATs) and PCR-based diagnostics to bind and differentiate particular virus RNA target sequences [96]. These assays are still among the most accurate and extensively used ways to diagnose SARS-CoV-2.

However, if one of the primer target sequences used in these tests is mutated, the primer may no longer be able to bind to the target, resulting in a false-negative result. Most NAATs are expected to have multiple genetic targets [106]. This means that even if a mutation occurs in one of the test target sites, the overall test should still function properly and produce accurate results. Mutations could also affect antigen and antibody tests, but the mutation would have to cause a change in the protein or physical structure of the virus being tested [107]. B.1.1.7, B.1.351, and P.1, the most well-studied VOCs (Table 3), all contain numerous mutations, as do others in the spike gene (S-gene) [108]. If any of these mutations influence the replication initiation sites or the protein/RNA structure of viral antigen targets identified during the antigen testing, the accuracy of diagnostic procedures may be impacted. Because of the existence of a 69/70 mutation in the S-gene, it leads to the deletion of two amino acid moiety, namely, N501Y and P681H. It is now commonly accepted that this is the case for the B.1.1.7 variation [109]. This mutation precludes the primers in various PCR tests with S-gene targets from binding to the S-gene target, particularly the Thermo Fisher Taq Path test [110]. Quantitative RT-PCR is useful for the detection of various mutations which are the causes of various variants, but it is highly specific to a certain amount of mutation. Whensoever new variant is observed, the entire probe setup needs to be updated and validated [111]. While the phenomenon is of rare occurrence, the FDA stated that the Cepheid test was inaccurate because of mutations in a single point [112]. In the initial screening, PCR-based on E484K and N501Y enhanced the sensitivity towards P.1 and B.1.351, but on further analysis, similar mutations were also present in other VOIs [113].

This causes S-gene target failure or S-gene dropout, which is a bad consequence for this target. The Thermo Fisher Taq Patch test, on the other hand, has three targets, two of which are still operating as expected [114]. There has been no impact on the overall accuracy of the test findings due to this built-in redundancy. Because most tests involve numerous genomic targets, it is reasonable to expect that the most widely used assays will remain accurate in the face of viral evolution. To present, there is no indication that S-gene mutations in other VOCs affect the performance of PCR testing, but it will be important for users of S-gene assays to keep an eye on this [115]. In the early phase of detection, sensitivity of antigen test appeared to be compromised and hence physicians relied on RT-PCR to a greater extent [116]. However, in further studies, it was concluded that the positive agreement of antigen test was higher than that of RT-PCR (90.0% and 73.7% respectively) [117]. Through comparison of viral culture, RT-PCR and antigen tests, sensitivity declined in the same series with 84%, 64% and 50%, respectively, when tested on the day of infection [36].

The mutations in other genes associated with other proteins not recognized as antigens, to date, do not appear to have influenced diagnostic testing [4,89,118]. Because most of the commercially available tests do not recognize the S-gene as a major target, it is critical to track the impact of mutations in all genomic areas [89]. Because of the significant risk of additional mutations in the S-gene, it should be avoided as a target in the future.

Besides the preponderance of RNA extraction in the diagnosis, other methods are also incorporated in order to perform the diagnosis. Saliva testing is accepted as a method which deducts the procedure of RNA-extraction. Along with selectivity and predictivity, it has add-on benefits such as a reduction in the need for any sophisticated machinery or transportation media, and hence, it is time saving [119]. However, the results rely on the method of extraction, nature of sample collection and the effectiveness of RT-quantitative PCR which varies [120].

## 7. Prediction Models for Diagnosis and Prognosis of COVID-19

Effective diagnosis and prognosis techniques for identifying the illness is the need of the hour to reduce the burden on the healthcare system along with providing the best healthcare facilities to the patients. Prediction models that incorporate numerous characteristics or attribute to identify people that have a high risk of being sick, or getting badly infected, can help the medical staff to prioritize the patients depending on their needs and the limited medical facilities available [121]. In the light of the demand to quickly and publicly communicate the key finding related to COVID-19 research to support the public healthcare response and save maximum lives, models starting from rule-based scoring systems to advanced machine learning models have been developed and published. Many of these prediction models are released without peer review in open access sources [122].

We selected and critically assessed various prediction models associated with the current pandemic. These prediction models were created to identify individuals in the mass community that is exposed to a high risk of getting admitted to the hospital due to the COVID-19 infection, to diagnose COVID-19 in patients who have symptoms, and to forecast their outcome (Table 3). All models showed good to exceptional predictive performances; however, they were all rated as having a high risk of bias, due to the culmination of poor reporting and procedural conduct for participant selection, statistical methods used, and predictor description [123,124].

The predicted execution protocols demonstrated a high/near-perfect capacity in detecting COVID-19. However, owing to poor reporting along with an artificial mix of patients with and without COVID-19, these models are prone to biases [125]. Ease of making medical decisions is the primary goal of such prediction models. As a result, it is important to set a target group for whom these predictions are beneficial and a representative dataset (ideally comprising consecutive patients) based on which these models are being constructed and verified [121]. The details of the target population should also be given, to evaluate the validated model’s performance. The WHO has recently created a novel data portal to motivate people for sharing anonymized COVID-19 clinical data to maximize new potential and allow individual participants to data meta-analyses. International and multidisciplinary collaboration in terms of data collection and model construction has been critical to fully exploiting the potential of these evolutions [126]. Various models such as Carr’s model, Qcovid models, Robust model, Random Forest, Gradient and RUSBoosting, the PRIEST score, ISARIC4C Deterioration model, Xie model etc. are incorporated in the prediction of diagnosis [127,128]. The Robust model predicts the urgency of decision-making criteria such as hospitalization, treatment, shielding and interventions [129]. The Qcovid model is involved in assuming the risk of mortality in COVID-19 and 4C mortality score for hospitalization [130,131]. The PRIEST score helps with decisions on whether or not to shift to the emergency compartment those patients who are suspected to have COVID-19 [132].

The diagnostic and prognostic models of COVID-19 show a fair to excellent discriminative performance. But these models, as discussed, are biased due to the selection of non-representative control patients, randomly removing patients that did not show predicted results by the end of the trials, and model overfitting [133]. Refer to Table 4 for the predicative study related to diagnostic tests. Therefore, their performance forecasts are prone to being too deceptive and optimistic. These are the issues that need to be addressed in future research. It is important to share data and tools for the development, characterization, and update of COVID-19-related prediction models [121,134].

The final models were either basic with four variables or complicated with 15 variables. To assist clinical usage, the authors suggested ratings that simplified the computation method and made clinical use easier [135]. The types of predictors included in the research varied (e.g., the included models comprised various combinations of clinical symptoms, socio-demographics (age and gender), laboratory or biological tests, and vital signs). This has implications in terms of clinical practice relevance [136]. Factors such as symptoms, gender, age, and contact tracing are of more value in models that are developed for triage patients on their first admission than laboratory or biological testing.

## 8. Deep Learning Techniques for the Diagnosis of Novel Coronavirus

COVID-19 has greatly threatened human civilization and emerged as the most hazardous disease of the decade [102]. With the advancement of contemporary technology in recent decades, inventive methods to aid illness diagnosis, prevention, and management have been developed that make use of smart healthcare instruments and facilities [137,138,139]. Comparing the use of different imaging techniques for diagnosis, though X-ray has the advantage of being economical and readily accessible, CT scanning is favored over X-ray because of its three-dimensional (3D) pulmonary imaging and advantageous adaptability features [140]. These imaging technologies have turned out to be highly critical in a fast diagnosis and thus in pandemic containment.

**Figure 2:**
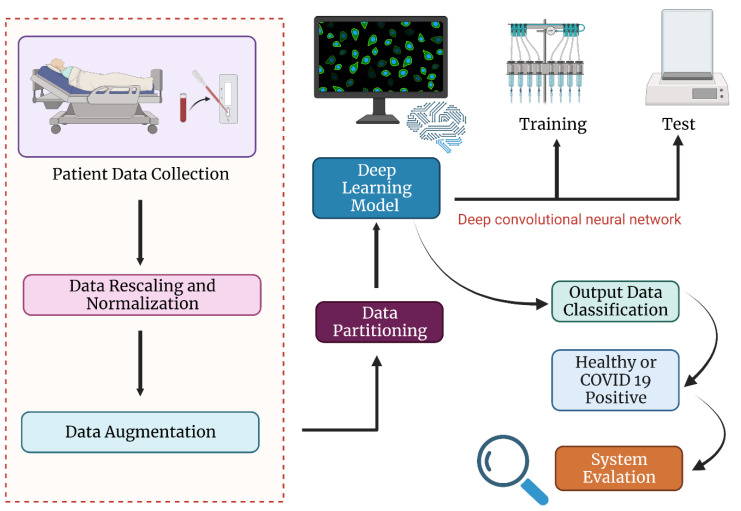
Deep learning techniques for the diagnosis of novel coronavirus.

Artificial Intelligence (AI), a rapidly emerging software-based technology in the field of medical image analysis, is also involved in the fight against the new coronavirus by effectively giving high-quality diagnosis findings while significantly decreasing or eliminating manpower [141,142]. DL and ML, two important fields of AI, have lately gained popularity in medical uses. DL-based assistance systems for COVID-19 diagnosis are being developed utilizing both CT and X-ray samples [143]. Pre-trained models are used for some systems, while others are introduced utilizing bespoke networks. Machine learning and data science, along with additional various domains, have been actively applied for coronavirus diagnosis, prognosis, prediction, and epidemic forecasting [144,145]. An overview of the novel mechanism for the diagnosis can be clarified in Figure 2. 

By learning from basic visualizations, DL systems may explain complicated situations. The capacity to learn accurate properties and representations of learning data in a more systemic way, where numerous layers are used consecutively, are the major qualities that increased the demand for DL approaches. DL algorithms are frequently utilized in medical systems, such as smart healthcare, medical image analysis, biomedicine, drug development, and so on [146,147]. DL-based systems, in general, comprise numerous processes, including data collection, data analysis, feature selection and classification, and performance evaluation [148].

For detection, diagnosis, classification, prediction, and prognosis of COVID-19, several DL approaches are being used [149]. Public datasets, along with individual datasets containing information about CT and X-ray scans, are being used for training and validating the models by several researchers. For defining the efficacy of methodologies employed in research, precision, specificity and sensitivity are the three main criteria [150]. Nevertheless, the area under the curve has been employed in various research to quantify the efficacy of the approach used to diagnose COVID-19.

The nominal measures to avoid the spread of disease and cause a pandemic situation include the early identification of COVID-19 patients, by employing deep-learning methods at a lower cost and consequence [151]. With the recent introduction of DL algorithms into radiology center equipment, the feasibility of obtaining a cheaper, faster, and safer diagnosis of this illness is possible. The employment of these strategies in diagnostics would provide significant help to radiologists in reducing human error along with support in decision making during critical situations [152]. This study lends credence to the notion that DL algorithms are a viable means of improving healthcare and enhancing the outcomes of therapeutic and diagnostic operations.

## 9. Futuristic Diagnostic Tools and New Research Lines

With the emergence of new variants of coronavirus, different waves of COVID-19, the exponential growth of positive cases in each wave, and the fatal nature of the virus, it is a need of the hour to develop rapid, cost-effective, and time-efficient tests, determining the severity of disease in a patient and achieve high accuracy in the results. Worldwide, the gold standard for detection of COVID-19 is RT-PCR, owing to its high accuracy; however, due to its time-consuming nature and requirement of skilled professionals for its operation, conducting millions of tests daily becomes tedious, thus delaying the results generation [22]. As an alternative, rapid antigen kits are used as in-field tests for point-of-care diagnostics. These tests can often be inaccurate and provide false-negative results, thus making confirmatory tests, such as RT-PCR, a need. Although precise in the determination of the presence of the virus, RT-PCR does not ascertain the disease severity, which increases the need for additional diagnostic tests, such as chest CT scanning [153,154]. With the emergence of AI and Machine learning in the pharmaceutical and biotechnology sectors, the technology is deployable for the detection of COVID-19-positive persons in crowded places and provides accurate and quick results. Along with that, the development of point-of-care testing helps in rapid in-field diagnosis of the disease and its progression, which can also be used as a bedside monitor for mapping the progression of the disease in critical patients [47,155,156,157]. Table 5 summarizes some of the futuristic technologies applicable in the diagnosis of COVID-19.

The above patents are cited from patent scope, a patent database of the World Intellectual Property Organization (WIPO, Geneva, Switzerland). Besides the already established strategies and novel detection kits being used worldwide, focus can also be given to extracellular vesicles, a specific type of vesicles responsible for carrying proteins, enzymes, organelles, receptors, mRNA, and microRNA from one cell to another. One of the types of extracellular vesicles is exosomes. Exosomes are derived from endosomes, and play a role in trans-cellular transport. Along with being the cargo for transport of proteins, enzymes, and other components, these were recently found to be carrying components of invading viral species such as SARS-CoV-2 from an infected cell to a healthy one, thus spreading the infection. The tetraspanin CD9 and TMPRSS2 molecules, present on the surface of exosomes, facilitate the entry of coronavirus in lung cells by cleaving the fusion glycoproteins [158,159,160].

As these exosomes are present in all types of body fluids, they can be used as biomarkers in the detection of a viral disease [160]. Different researchers have found out that in pathological conditions, such as Tuberculosis, Hepatitis A and C, and HIV-AIDS, the transfer of the pathogen from the infected cell to a healthy one takes place via the help of exosomes [160,161].

There is a need for the identification of reliable biomarkers that we can use in forming successful detection strategies for infectious diseases such as COVID-19. Platelet extracellular vesicles have been reported to increase significantly in a patient suffering from mild and severe COVID-19 as compared to a healthy individual, upon their detection in platelet-free plasma. Detection of pEVs (exhibiting phosphatidylserine on the outer side of the membrane (“eat-me” signal)) can help in the determination of the level of organ damage and coagulation of blood [162]. Elletra et al. reported for the first time regarding the presence of COVID-19 in the exosomes of patients using proteomics, demonstrating that exosomes can be involved in the spread of infection throughout the different cells of the body [163]. Developing a diagnosis of COVID-19 by exosomal detection of the virus can help in the determination of the severity of the disease along with the accurate detection of the disease in the symptomatic patient.

Diagnosis of exosomes carrying the components of SARS-CoV-2 can be performed by using fluorescent or bioluminescent dyes, which can label the protein of interest. Detection can also be performed using CT scanning, positron emission tomography (PET) scanning, or magnetic resonance imaging (MRI). Besides exosomes constituting an excellent target for the development of diagnostic tools for COVID-19, there is a need to identify standard biomarkers for the detection of exosomes and formulation of strategies for the development of diagnostic tools for COVID-19 based on exosomes [164]. Convalescent plasma-derived exosomes are the niche sector for diagnosis as well as therapeutics of COVID-19 [165,166].

## 10. Conclusions

During the beginning phases of the COVID-19 pandemic, a significant number of diagnostic companies were proactively involved in the design, development, validation, verification, and deployment of diagnostic tests. Hundreds of molecular tests and immunoassays were created quickly, albeit many are still awaiting clinical validation and formal approval. However, greater test refinements and substantial molecular epidemiological confirmation, including official FDA certification, are still required. Furthermore, biobanks and the follow-up of actual patients are still inadequate, and AI and machine learning techniques for data interpretation must be created and used. To battle present and future pandemics, there must be worldwide unity in terms of test availability, and more critically, infection control and diagnostic treatments must be tightly interwoven. Diagnostics should inform therapy selection and follow-up on therapy success. It is important to highlight that deep learning algorithms combined with imaging modalities provide only limited information regarding sick individuals. The current study does not imply that deep learning approaches can replace the function of physicians or clinicians in clinical diagnosis. Deep learning specialists are hoped to collaborate pro-actively with radiologists and medical professionals in the near future to give adequate support systems for diagnosing COVID-19 infections, particularly in the early stages of the disease, or evaluating the level of severity of the infection. As per our point of view, AI-based diagnostic techniques and 3D deep learning techniques could possibly develop into one of the most accurate and sensitive methods for diagnosis in the near future. This is because these methods are easily modified to detect any changes in the antigenic structure. They are sensitive enough to detect even a minute quantity of antigen, which is crucial in early detection. In addition, they are easy to operate and rely on machinery, which helps to reduce human error.

## Figures and Tables

**Table 1:** Comparing sensitivity and predictivity of different sampling areas.

Parameters	Nasopharyngeal Swabs	Pooled Nasal and Throat Swabs	Saliva	Nasal Swabs	Reference
Sensitivity	97%	68%	85%	86%	[21]
Predictive Value	-	97%	93%	96%	[21]

**Table 2:** Summary of prominent diagnostic techniques for the detection of SARS-CoV-2.

Test	Principle	Target	Reliability	Specificity	References
Nucleic acid-based tests	Identification and amplification of the viral genetic material	Complementary DNA	The most reliable technique	99% (of RT-LAMP)	[24,55,56]
Computed Tomography (CT) scan	X-ray measurements of infected lungs	Lungs	Results may coincide with other viral pneumonia	25–80%	[39,57]
Protein-based techniques	Antigen–antibody interactions where either the antigen or the antibody from the patient’s sample reacts with the respective antibody or antigen present on the test kit	Spike proteins, glycan proteins, and nucleocapsid proteins peculiar to SARS-CoV-2	The sensitivity and specificity of the test were improved by kits with combined efficiency to detect IgA, IgG, and IgM	90.63%	[46,47,49,58]
CRISPR/Cas system	DNA/RNA targeting is employed in conjunction with the amplification process for the simultaneous detection and quantification of the viral load in the isolated samples	DETECTR—N and E genes SHERLOCK—S gene and Orf1ab of SARS-CoV-2	Highly specific detection of SARS-CoV-2 with sensitization efficiency of 2 copies per sample in time duration of 50 min	95% (positive prediction rate)	[59,60,61]

**Table 3:** Viral Mutations and their prevalence across the globe [5,6,8,97,98,99,100,101,102,103,104,105].

Variant	First Identification’s Location	Date	Mutations Observed	Clinical Changes			Number of Countries Reporting Variant
				Transmissibility	Virulence	Antigenicity	
B.1.1.7	United Kingdom	20 November	NS01Y	Increased	Increased	No Change	84
B.1.351	South Africa	21 December	N501Y, E484K	Increased	No evidence	Investigation stage	42
P.1.501Y	Brazil	21 December	NS01Y, E484K K417N	Under Investigation	Unknown	No Change	21

**Table 4:** An overview of COVID-19 infection diagnosis and prognosis prediction models.

Predictors	Validation Type	C Index (Performance)	Risk Assessed by PROBAST
Diagnosis			
Temperature Heart rate Diastolic blood pressure Systolic blood pressure Basophil count Platelet count Mean corpuscular hemoglobin content Eosinophil count Monocyte count Fever Shivering Shortness of breath Headache Weariness Sore throat Fever categorization	Temporal validation	0.94	+++
Fever, close contact history, CT evidence of pneumonia, neutrophil to lymphocyte ratio, highest body temperature, sex (age, meaningful respiratory syndromes)	Training test split	0.97	+++
Uric acid, triglyceride, serum potassium, albumin/globulin, 3-hydroxybutyrate, serum calcium, age, activated partial thromboplastin time, red blood cell distribution width SD, serum potassium	External validation	0.87	+++
**Prognosis**			
Combination of demographics, signs and symptoms, laboratory results, and features derived from CT images	Internal Validation	0.95	+
Age, LDH, lymphocyte count, SPO_2_	External validation	0.99	+
Clinical scorings of CT images	External validation	0.91	+

+++ Very High. + High. CT = Computed Tomography Imaging; SD = Standard Deviation; LDH = Lactate Dehydrogenase; SPO_2_ = Percent Oxygen saturation.

**Table 5:** Futuristic Diagnostic tools and new research in the field of diagnostics of COVID-19.

Sr. No.	Test/Kit	Biomolecules/Parameters Used	Target Molecule	Lower Detection Limit/Detection Parameters	Detection Time	Principle/Technology Used	International Patent Application Number
Artificial Intelligence, Machine learning, deep learning neural network, and radio imaging-based diagnostic tools							
1	AI-based diagnosis device	Vitals of normal person and patients body temperature respiration rate, respiration volume, etc.	Physical screening of subject	The physical distance between the individual and the acquisition device of 60-2 cm	30–120 s	Artificial Intelligence-based non-contact diagnosis of COVID-19; Uses devices such as a thermal camera for subject screening; Data processing device used to assess the individual; Results are displayed on a display device.	PCT/EP2021/062048
2	Convulsion Neural Network and chest radiography	CNN made from a database of chest X-ray images of COVID-19 positive patients	Chest X-ray of subject	-	-	A database of chest X-ray images of patients in constructed; Sample data and training data modules are formed; Training and learning modules are formed; CNN is formed using a deep learning model–this detects COVID-19 using X-ray images of patients.	2021104727
3	Diagnosis using spectroscopy and artificial intelligence	Saliva or nasopharyngeal swab	Proteins, microbes, and antibodies	-	-	COVID-19 patient samples are analyzed using infrared spectroscopy (FTIR or ATR-IR); Spectral vibrations are identified in COVID-19 positive samples; Using AI diagnostic samples can be identified for the presence of viruses without the need for reagents.	PCT/BR2021/050234
4	3D deep learning network	Deep learning of 3D CT-scan	3D CT scan of the patient	-	-	Detection of COVID-19 pneumonia, non-COVID-19 pneumonia, non-pneumonia abnormal or normal condition of the lungs using deep learning of 3D CT-scans of lungs; The technology can be used to monitor the disease progression.	17067181
5	A real-time diagnostic system using machine learning	Vital parameters such as Heart rate Respiratory sounds Oxygen saturation Body temperature	-	-	Real-time measurement	The system measures the respiratory sounds, oxygen saturation, and body temperature of a person or COVID-19 positive patient in real-time; Uses machine learning software to detect abnormality and disease progression.	202041030100
6	Deep learning-based system	CT images of the lungs and the infection within	Chest CT scan	-	-	Determining the disease burden and seriousness by using deep learning of segmented CT images of lungs and the disease within it.	2021100007
7	Artificial Neural Network based diagnosis using chest images	Database chest images	Chest images such as X-ray	-	-	First neural network in which a synthetic COVID-19 chest image is made using a database of COVID-19 patient chest images; This is transferred into a second neural network to establish learning of different prognostic images; Target chest image is inputted in a second neural network to conclude the presence and severity of COVID-19 infection in the patient;	PCT/KR2021/006704
Point-of-care diagnostic tools							
8	Real-time POC Disease detection based on electromagnetic resonance	Human body	EM Frequency of the virus or its genome	30 s (or less)	Electromagnetic frequency	Electromagnetic radiation is used to detect the electromagnetic frequency of a particular disease or its nucleotide; The subject can stand in a walk-in detector chamber wherein an EM frequency of a particular spectrum is charged on the body; The reflected electromagnetic frequency is detected and compared with that of the disease from the database for detecting the presence of disease.	17220769
9	Point-of-care Serology test	Blood plasma Saliva sample Nasopharyngeal swab	IgG or IgM	Z-score of 5 or greater	<30 min	Micro-array detection of antibody in a serological sample by binding to it; The result will be compared in form of a z-score with the data based on the connected cloud server.	17083113
10	Point-of-care test	Nasopharyngeal swab	RNA-dependent RNA polymerase gene of nCOVID-19	-	~12 min	A denatured sample is annealed with a primer to which the gold nanoparticle solution can bind; If COVID-19 antigen binds with primer, gold nanoparticles are unbound and thus aggregate and turn the color from red to blue; In absence of antigen, primer binds with nanoparticles and prevents the agglomeration, the color remains unchanged.	202011018132
PCR, Immunoassay, and chromatography-based diagnosis							
11	Genomic profiling of body micro-flora in healthy and disease conditions	Oligonucleotides and primers	Micro-organisms	-	-	Levels of certain bacteria/viruses in patient’s stool samples are detected; Its correlation with disease severity, recovery time, development of pneumonia, need for intubation, and prediction of death can be performed.	PCT/CN2021/090488
12	The chromatographic rapid detection immunoassay device	Spike protein S1 subunit (RBD) in body fluid such as Blood Urine Saliva	IgM, IgG antibodies	A cut-off value of 25 and above	10–15 min	Ligands are bound on sorbent strips which will detect IgM or IgG against SARS-CoV-2 produced in the sample.	PCT/US2021/031552
13	Microbeads for detection of the viral genome	Body fluids including Blood Serum Mucus Saliva Urine	Viral genome	Fluorescence detection	1–4 h	Fluorescence emitting microbeads are arranged in an array; Initiation primer is attached to the beads; Second fluorescence is emitted by detection sequence and presence or absence of viral; Nucleic acid is a detected amount of fluorescence.	PCT/US2021/025239
14	A lateral flow assay device	Oral swab Nasal swab Sputum or saliva	Whole virus	Red to purple	<3 min	The kit comprises gold nanoparticles embedded on an absorbent pad; They are conjugated with a peptide that can specifically bind to the S1 spike protein of coronavirus; The presence of the virus is detected colorimetrically when the test region turns red to purple.	PCT/IN2021/050583
15	Detection using CRISPR-CAS9-mediated guide RNA	Nasopharyngeal swab Mucosa, saliva Blood Urine Feces Any biological fluid	SARS-CoV-2 RNA	-	-	Cas-9-mediated guide RNA specific for Open reading frame of amplified coronavirus genome from the sample is used; Detection is performed using the fluorescent or chemiluminescent assay.	PCT/KR2021/003429
Diagnostic tools without the need for reagents							
16	Electronic nose	Exhaled air	Pheromones in breath	e-map display	Few minutes	Diagnosis of COVID-19 before the appearance of clinical symptoms using an electronic nose-like device which detects pheromones in breath.	PCT/RU2020/000265
17	Artificial nose	Breath sample	Volatile organic compound	The difference in VOC profile level by 5% or more compared to control	2–3 min	Ligands used to detect volatile organic compounds from a person’s breath; A probe a.k.a. “artificial nose” is used; compared with the VOC profile of a healthy person; The technology helps in detecting the presence of virus and viral load; Allows the determination of the stage of the disease before the onset of symptoms.	PCT/IL2021/050451

## Data Availability

Not applicable.
